# Delivery of a fibrin-binding hemostatic polymer ameliorates neurovascular damage and neural tissue loss after traumatic brain injury

**DOI:** 10.1126/sciadv.adw7425

**Published:** 2025-07-18

**Authors:** Qinghua Han, David Brenes, Kevin W. Bishop, Trey J. Pichon, Melissa Ling, Garrett D. McPheron, Nathan J. White, Suzie H. Pun, Jonathan T. C. Liu, Drew L. Sellers

**Affiliations:** ^1^ Department of Bioengineering, University of Washington, Seattle, WA 98195, USA.; ^2^ Department of Mechanical Engineering, University of Washington, Seattle, WA 98195, USA.; ^3^ Department of Molecular Engineering and Sciences Institute, University of Washington, Seattle, WA 98195, USA.; ^4^ Department of Emergency Medicine, University of Washington, Seattle, WA 98195, USA.; ^5^ Department of Chemical Engineering, University of Washington, Seattle, WA 98195, USA.; ^6^ Department of Laboratory Medicine and Pathology, University of Washington, Seattle, WA 98195, USA.; ^7^ Institute for Stem Cell and Regenerative Medicine, University of Washington, Seattle, WA 98109, USA.

## Abstract

Traumatic brain injury (TBI) often induces blood leakage into brain tissues, which causes further tissue loss after the initial injury. To mitigate this secondary injury, we hypothesized that delivery of a fibrin-binding hemostatic polymer, PolySTAT, would act as a molecular patch and ameliorate brain vessel damage following TBI. We developed a three-dimensional (3D) pathology and analysis workflow to quantify the effects of PolySTAT versus a control polymer, PolySCRM, on neurovascular networks and neural tissue in whole mouse brains. Using a panel of fluorescent probes, our 3D pathology pipeline revealed that PolySTAT treatment preserves neurovascular density and function, reduces hypoxia and blood extravasation, and reduces brain tissue loss after TBI. To further corroborate the 3D microscopy–based findings, gene expression analyses show that PolySTAT attenuates the expression of inflammation and reactive gliosis biomarkers. These findings support future translational investigation of intravenous PolySTAT as an early post-injury therapy to mitigate neural tissue loss after TBI.

## Teaser

3D pathology shows that a fibrin-binding polymer increases neurovascular network and neural tissue sparing after TBI.

## INTRODUCTION

Each year, more than 50 million people worldwide suffer a traumatic brain injury (TBI), with symptoms ranging from mild, short-term concussions to severe, permanent disabilities and even death (*1*, *2*). As a consequence of a sudden blow to the head, TBI compromises the tightly regulated brain neurovasculature that maintains a critical blood-brain barrier (BBB). Upon injury, fluid and blood proteins leak into the brain tissue, which induces brain swelling and inflammation (*3*). Damage to blood vessels also impairs oxygen delivery to the brain, which results in a low-oxygen (hypoxic) environment. As a result, neurovascular leakage and dysfunction create a dangerous feedback loop of molecular signaling that further increases the leakiness of surrounding blood vessels (*4*). The blood that extravasates into the brain is toxic to the surrounding tissue, resulting in rapid neural cell loss in a cascading chain reaction of destruction that makes TBI so devastating to patients (*5*–*9*). Although the injured vasculature attempts to self-repair, vessel dysfunction can persist long-term after TBI, resulting in exacerbated neurodegeneration (*10*). As a result, TBI survivors, especially the approximately 20% diagnosed as moderate to severe cases, have an increased risk of developing Alzheimer’s disease, Parkinson’s disease, and chronic traumatic encephalopathy (*11*, *12*). Thus, fast-acting strategies that facilitate neurovasculature stabilization after TBI could reduce brain damage, stem the amplifying cascade of neuroinflammation triggered by injury, and improve long-term cognitive outcomes for TBI victims.

When neurovascular damage occurs, fibrin is deposited within the brain at wound sites (*13*). We previously developed an injectable peptide co-polymer, PolySTAT, that binds to and cross-links fibrin at wound sites to reduce blood loss after trauma (*14*). In a rat femoral artery (leg) injury model, we demonstrated that intravenous PolySTAT significantly staunched bleeding and improved survival (*15*). Both PolySTAT and the cognate fibrin-binding peptide have been shown to only accumulate at sites of fibrin deposition, with a functional binding half-life of ≥1 hour post-injection, and rapid renal clearance (*13*, *16*, *17*). In recent work by others, platelet-like microgel particles that bind to fibrin were administered after TBI to reduce coagulopathy and blood-brain barrier (BBB) permeability. When administered 2 hours post-injury, these platelet-like particles increased tissue sparing and reduced inflammation 7 days post-injury (*18*). We therefore hypothesized that administration of PolySTAT early after TBI could stabilize the neurovasculature, reduce bleeding into the brain, and minimize inflammation to mitigate ongoing injury to neural tissue. However, a major challenge in testing therapies like PolySTAT is the lack of accurate and quantifiable brain imaging modalities.

Conventional imaging modalities, such as computed tomography (CT) and magnetic resonance imaging (MRI), offer an indirect assessment of morphological and anatomical changes caused by TBI. These modalities typically lack the resolution to image and quantitatively assess capillary networks (*19*, *20*). To address this limitation, advanced three-dimensional (3D) microscopy techniques have been developed for high-resolution imaging of brain tissues in murine models. Microscopic examination is crucial for assessing how therapeutic interventions affect neurovascular histomorphology and function after TBI. Imaging techniques like two-photon microscopy (*21*) and light-sheet microscopy (*22*–*24*), coupled with tissue-clearing protocols such as iDISCO (*25*), CUBIC (*26*), and CLARITY (*27*), have enabled detailed imaging of entire brain structures. These 3D methods offer a superior alternative to traditional microscopy pipelines that rely on labor-intensive processes such as serial sectioning (*21*), which can introduce artifacts that skew volumetric analyses. In addition to generating 3D reconstructions of brain vasculature and tissue architecture, which provide insights into neurodegeneration and other pathophysiology (*28*–*30*), a nondestructive whole-brain imaging workflow allows for downstream molecular analysis of selected tissue volumes (*31*, *32*).

Despite recent imaging innovations, substantial challenges remain in analyzing large 3D datasets, especially in the context of TBI. Analysis pipelines often require manual interventions and annotations for proper image registration, segmentation, and data analysis. User bias can thus introduce variability that inadvertently skews data collection and study results (*33*). Fully automated and rapid computational methods offer the ability to image larger numbers of samples in different experimental cohorts and across various laboratories with high reproducibility and minimal human bias. Toward this end, recent pipelines have incorporated standardized brain atlases, like the Allen Brain Atlas (*34*), to spatially register brains and enable automated analyses of anatomical regions from different mice (*35*–*38*). However, these brain atlases require precise image-based registration, which is challenging due to variations in brain size and morphology across individual subjects, especially if their brains are injured and deformed as in the case of TBI (i.e., the brain atlases are for “normal” brains) (*39*). Additionally, existing analysis pipelines have mostly focused on specific structures [e.g., neurons (*37*, *38*) or vessels (*35*, *36*)] in relatively normal mouse brains and are not designed to quantify multiple features associated with brain injury or neurodegeneration, such as hypoxia, BBB permeability, and tissue loss.

Here, we used a customized open-top light-sheet (OTLS) microscope to volumetrically image whole mouse brains with capillary-level resolution, providing an unbiased and comprehensive analysis of TBI’s effects on neurovasculature and brain tissue. We also developed a multi-channel 3D image analysis pipeline to quantify vascular density/volume, tissue hypoxia, and blood extravasation as a function of distance from the surface of the TBI-induced lesion (i.e., the cortical surface after TBI-induced tissue loss), as well as the extent of overall tissue loss. Our analysis can accommodate variable deviations in brain anatomy between subjects, making it particularly well suited for studying brain injury compared with methods that require spatial registration to a normal brain atlas. As a demonstration of our imaging and analysis pipeline, we show that intravenous PolySTAT treatment following TBI preserves functional brain vasculature; reduces blood extravasation, tissue loss, and hypoxia; and decreases the expression of genes associated with inflammation and reactive gliosis in the brain. We provide an open-source tool for investigating damaged brains that are anatomically unpredictable, showcasing the ability to quantify pathological changes associated with TBI and evaluate the efficacy of a therapeutic intervention. This pipeline has broad potential applicability to other injury models where individualized analyses of diverse pathological and anatomical changes are essential.

## RESULTS

### Whole-brain 3D fluorescence microscopy workflow for analysis of brain injury

**Fig. 1.**
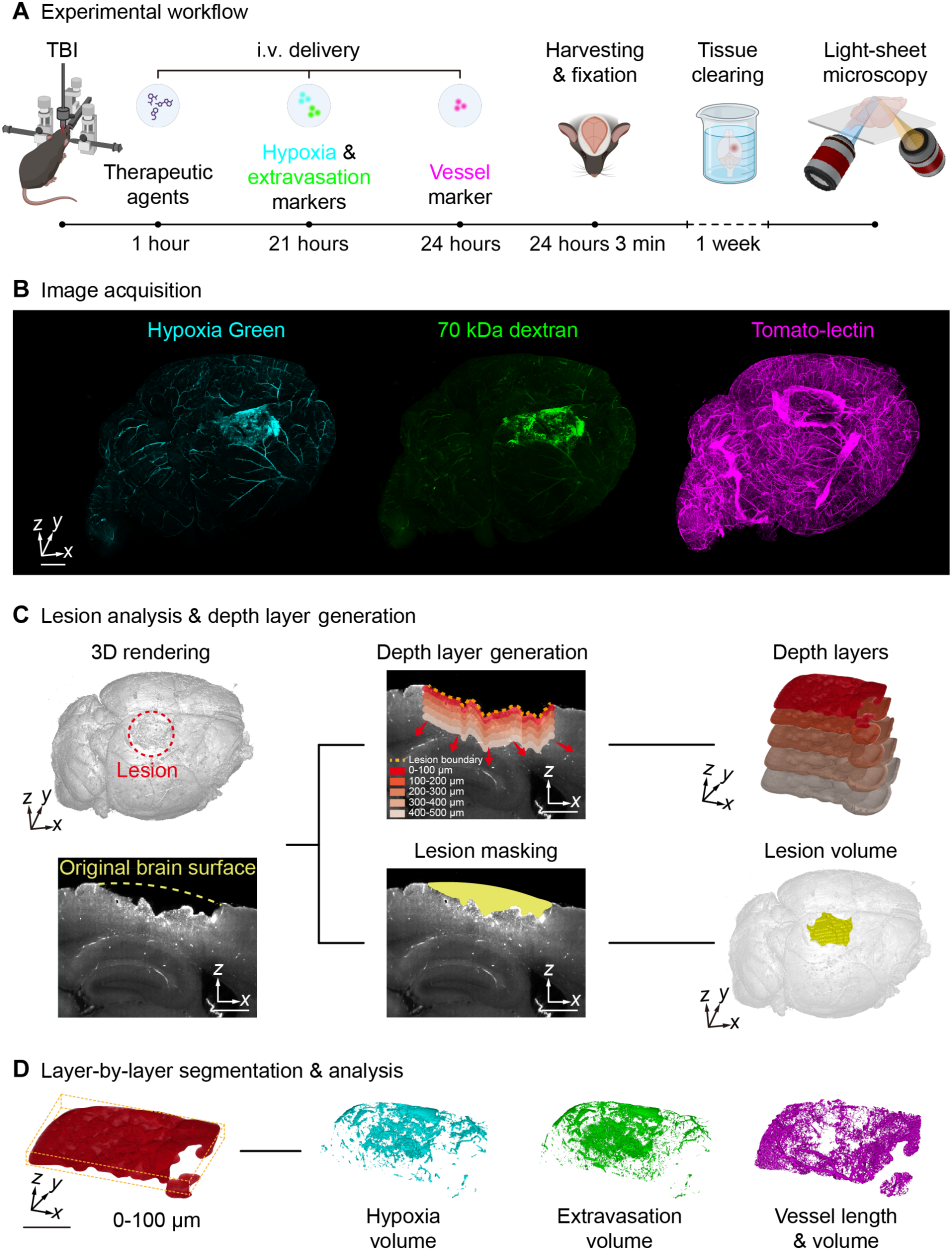
Experimental approach and 3D image processing workflow. (**A**) Schematic and timeline of the experimental workflow to evaluate how administration of a hemostatic polymer (1 hour post-injury) affects neurovascular networks, hypoxia, and extravasation following CCI-TBI. Intravenous injection (i.v.) of hypoxia and extravasation markers occurred 21 hours post-injury, followed by vessel labeling at 24 hours post-injury. Three minutes after vessel labeling, mice were euthanized to harvest brain tissue, which was processed by a 7-day iDISCO tissue-clearing protocol. (**B**) Open-top light-sheet (OTLS) microscopy datasets of three fluorescence indicators. Scale bar, 1 mm. (**C**) Overview of the image processing workflow for quantifying lesion volumes and for defining/segmenting depth layers at various distances from the lesion. Scale bar, 500 μm. (**D**) Layer-by-layer quantitative analysis of vasculature, hypoxia, and extravasation following 3D segmentation of fluorescently labeled tissue structures. Scale bar, 1 mm. Created in BioRender. Han, Q. (2025) https://BioRender.com/jxsbpkn.

The damage from TBI triggers a multitude of pathologies in brain tissue regions far beyond the site of direct impact or primary injury. Therefore, we developed a computational pipeline to assess injury markers throughout whole brains, focusing especially on quantifying damage as a function of distance from the original injury site/lesion. Our experimental workflow is shown in Fig. 1A. A small (2-mm-diameter) craniotomy is performed on mice. Next, a contusion device is placed on the top of the brain to create a controlled cortical impact (CCI)–TBI. We injected mice 1 hour post-injury with either PolySTAT or a control polymer (PolySCRM), which has a similar composition to PolySTAT but lacks fibrin-binding abilities (i.e., containing scrambled, nonbinding peptides). In separate studies, we confirmed that PolySTAT, but not PolySCRM, homed to the brain injury site after intravenous injection (fig. S1). Sham animals, which only received a craniotomy but no CCI-TBI, were also included to account for tissue deficits that result from the craniotomy or other minor sources of tissue damage introduced throughout the experimental time course. Twenty-four hours post-injury, brains were harvested and fixed for imaging and analysis. We selected three metrics to evaluate brain vasculature health: vascular structure, vascular integrity, and tissue hypoxia. To quantify these parameters, before euthanasia and brain harvesting, we injected mice intravenously with three fluorescent indicators: tomato-lectin to visualize vasculature; Texas Red–labeled dextran 70 kDa, which can only extravasate into the tissues if the vasculature is compromised; and Hypoxia Green, which fluoresces in low oxygen environments (Fig. 1A) (*40*–*42*).

Excised whole mouse brains were optically cleared with a standard iDISCO protocol using ethyl cinnamate for refractive index matching, followed by 3D OTLS microscopy (Fig. 1B) (*43*). Raw OTLS microscopy datasets were processed to create a stitched and fused 3D dataset (i.e., a continuous volumetric representation of the imaged mouse brain; fig. S2, A to C). The background autofluorescence within the dextran channel was used to identify and quantify regions containing brain tissue. We then applied an image filter to computationally define the lesion boundary, which comprises the CCI-TBI site and the surrounding tissue lost as a consequence of the injury. Next, we implemented a computer-vision dilation algorithm to segment 3D conformal layers at increasing distances emanating away from the lesion boundary (Fig. 1C and fig. S3A). Finally, various injury markers were quantified within each of these conformal depth layers to quantify damage attributable to TBI (i.e., a layer-by-layer analysis) (Fig. 1D and fig. S3B).

### PolySTAT enhances neurovascular sparing after CCI-induced TBI

**Fig. 2.**
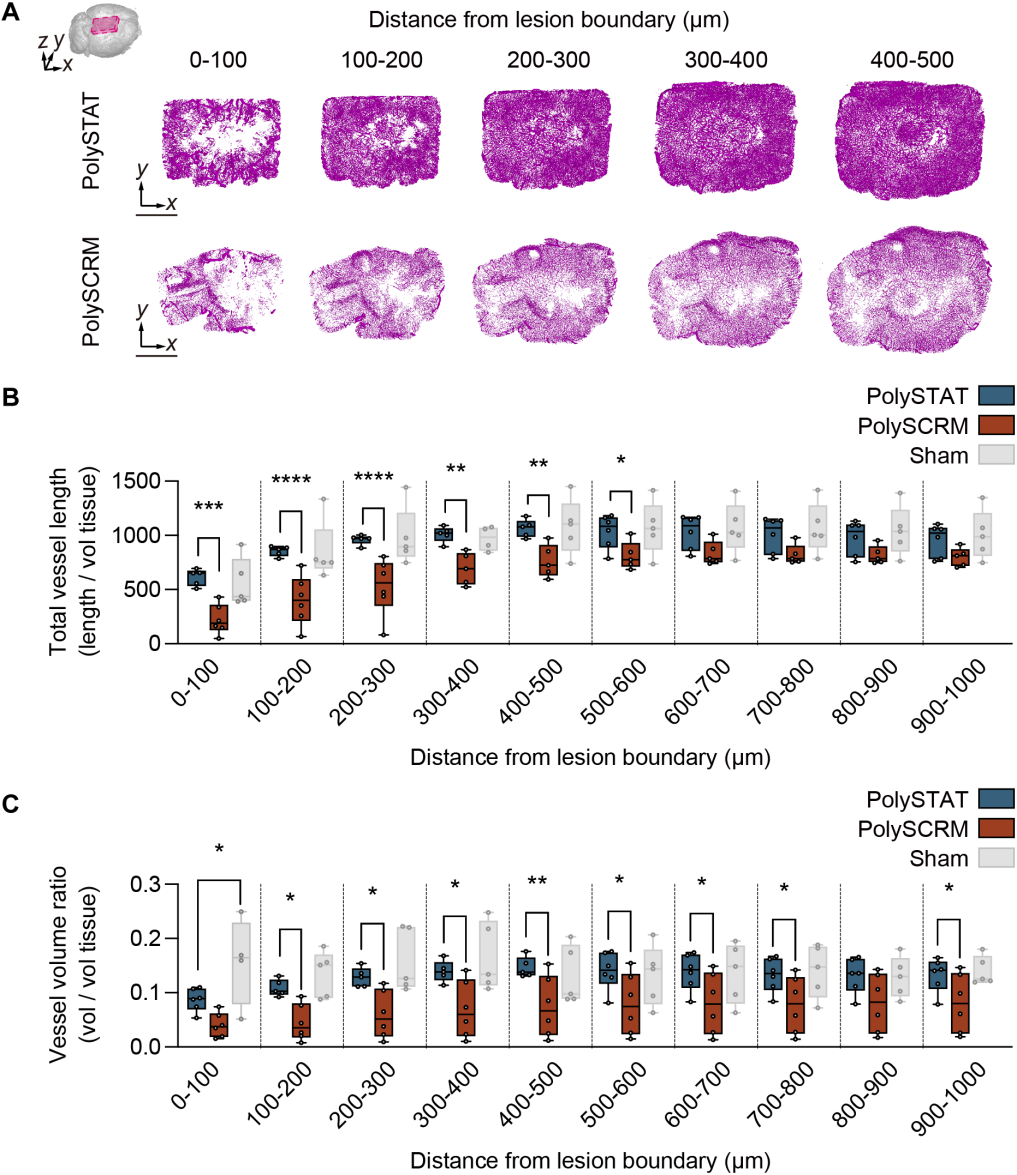
Effects of PolySTAT on the neurovascular network. (**A**) En face views of volume-rendered vascular structures up to 500 μm from the lesion boundary for one brain from each of the PolySTAT- and PolySCRM-treated groups. Scale bar, 1 mm. Box plots illustrate that the PolySTAT-treated mice (*n* = 6) have preserved overall vessel length (**B**) and vessel volume (**C**) compared to the PolySCRM-treated mice (*n* = 6). Uninjured sham mice (*n* = 5) served as controls (statistical comparisons to PolySCRM-treated mice are not shown). *P ≤ 0.05; **P ≤ 0.01; ***P ≤ 0.001; ****P ≤ 0.0001 by one-way analysis of variance (ANOVA) with Fisher’s least significant difference (LSD) test.

Since vascular damage underlies much of the clinically observed disability in TBI victims, we injected a fluorescent tomato-lectin to label and quantify the effects of TBI on the neurovascular network in TBI brains treated with PolySTAT or PolySCRM, as well as in uninjured shams. We then implemented our layer-by-layer analysis in conjunction with a Hessian-based Frangi vesselness filter (*44*) to computationally identify and isolate lectin-labeled blood vessels throughout the brain (fig. S4A). After vessel isolation and segmentation within the cortex, we processed each segmented cortical volume using skeletonization and Euclidean-distance algorithms to extract vessel centerlines and radii (*45*). We then calculated vessel lengths and volumes of the neurovascular network (Fig. 2A and fig. S4, B and C). Analysis of 100-μm-thick conformal layers emanating from the lesion boundary revealed that the total vessel length per tissue volume was 28% to 174% greater in PolySTAT- versus PolySCRM-treated animals at distances of up to 600 μm from the lesion boundary (Fig. 2B; total vessel length measurements for sham versus intact animals are reported in fig. S7A). Moreover, the total vessel length in PolySTAT-treated animals was comparable to that of uninjured sham animals that received a craniotomy without CCI-TBI. Combined, these studies demonstrate that PolySTAT reduces vessel loss and preserves vascular network integrity and complexity after TBI.

Previous studies have demonstrated that blood vessels are lost and that vessel diameters decrease after TBI, which has the potential to contribute to hypoxia and to cause further tissue damage (*46*, *47*). Thus, we next analyzed vessel diameters and lengths to determine the volume of the vascular network in PolySTAT- and PolySCRM-treated brains, as well as uninjured sham brains. We normalized the vascular volume by tissue volume (mm^3^/mm^3^) and performed a layer-by-layer analysis from the lesion boundary. We found that neurovascular volume increased by 78% to 143% within 600 μm from the lesion boundary in PolySTAT-treated animals compared to PolySCRM-treated animals (Fig. 2C; vessel volume measurements comparing sham and intact animals are reported in fig. S7B), and that intravenous PolySTAT increased vessel volumes by 65% far beyond the lesion boundary (≥1 mm). Thus, PolySTAT treatment mitigates the vascular dysfunction that propagates from the primary TBI lesion. Moreover, at depths greater than 100 μm from the cortical surface, neurovascular volume did not differ significantly in PolySTAT-treated TBI animals compared to sham animals. Moreover, the neurovascular volume in PolySCRM-treated TBI animals was reduced by 37% to 67% compared to sham animals throughout the cortex. Combined, these data demonstrate that PolySTAT treatment enhanced neurovascular sparing in brain tissue when administered acutely after CCI-TBI.

### PolySTAT protects vascular integrity and reduces hypoxia after injury

The effects of vascular damage that evolve after TBI have been demonstrated in several animal models (*48*). BBB disruption results in extravasation of circulating proteins that promote neuroinflammation and neural cell death (*49*). The loss of blood vessels also impairs oxygen delivery, which increases hypoxia and further exacerbates tissue loss (*50*, *51*). Therefore, we examined whether PolySTAT reduces hypoxia and macromolecule extravasation into the brain parenchyma after TBI. For TBI animals treated with either PolySTAT or PolySCRM, as well as uninjured sham brains, we systemically injected two indicator dyes: (i) a live-cell permeable compound that becomes fluorescent in low oxygen environments (Image-iT Green) to measure hypoxia, and (ii) a fluorescently tagged high–molecular-weight dextran (70 kDa) to measure vascular extravasation.

**Fig. 3.**
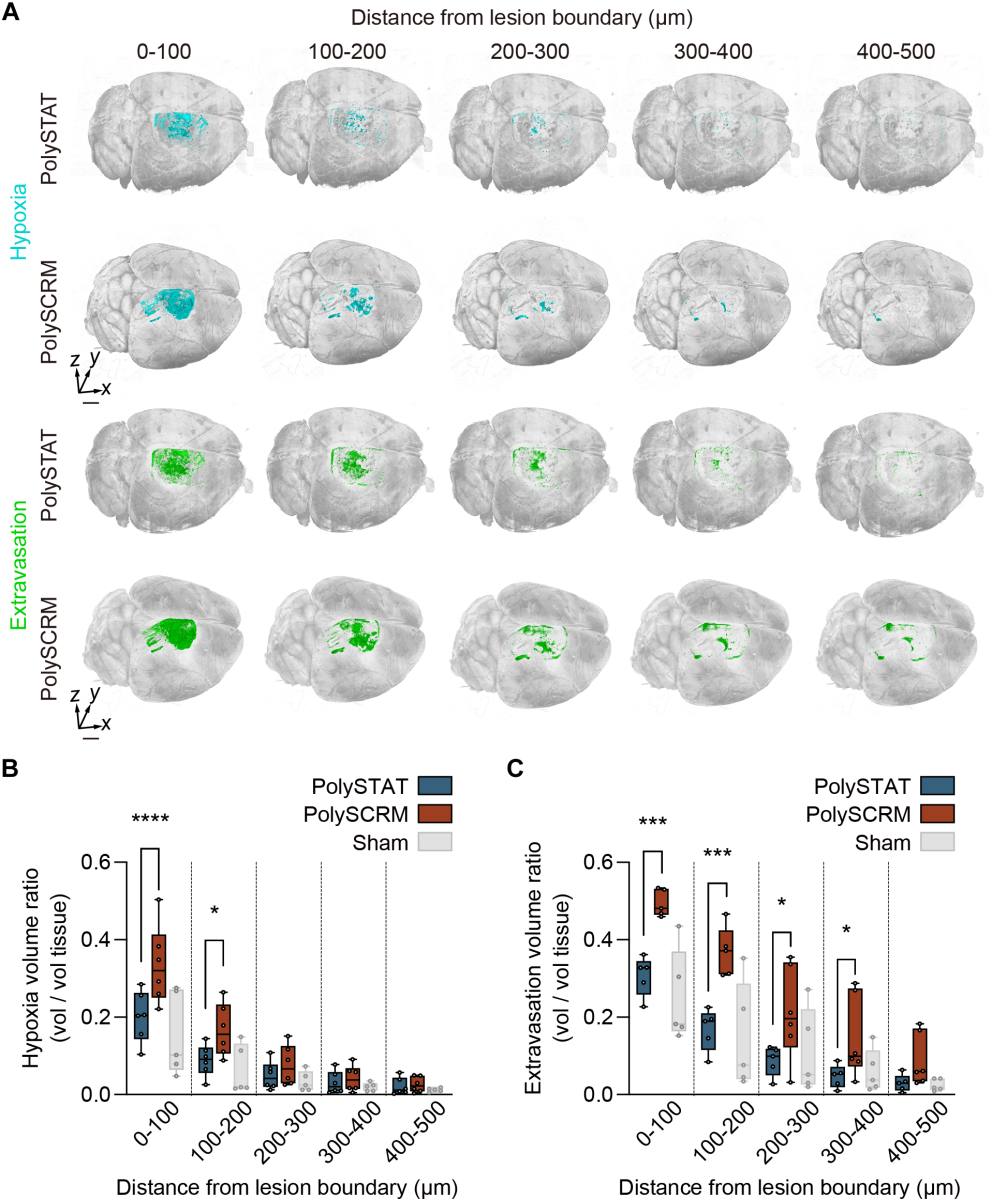
Effects of PolySTAT on the extent of hypoxic stress and blood extravasation. (**A**) 3D volume rendering of hypoxia masks (cyan) and extravasation masks (green) for one brain from each of the PolySTAT- and PolySCRM-treated groups. Scale bar, 1 mm. Box plots show that PolySTAT-treated mice (*n* = 6) have reduced hypoxia volume (**B**) within the first 200 μm and reduced extravasation volume (**C**) within the first 400 μm from the injury site, compared to PolySCRM-treated mice (*n* = 6). Uninjured sham mice (*n* = 5) served as controls (statistical comparisons to PolySCRM-treated mice are not shown). *P ≤ 0.05; ***P ≤ 0.001; ****P ≤ 0.0001 by one-way ANOVA with Fisher’s LSD.

Whole-brain images revealed that hypoxia and vascular extravasation occurred predominantly near the injury site (Fig. 3A), which is likely due to neither dye being able to cross the BBB. Nevertheless, application of a custom denoising algorithm revealed distinct differences in hypoxia and extravasation signal localization and tissue distribution between PolySTAT- and PolySCRM-treated animals (fig. S5, A to D). Analysis within conformal layers emanating from the lesion boundary demonstrated that hypoxia was reduced by 40% to 47% within the first 200 μm from the lesion boundary in TBI animals treated with PolySTAT versus PolySCRM (Fig. 3B; hypoxia measurements for sham versus intact animals are reported in fig. S8A). Similarly, 70-kDa dextran extravasation was reduced throughout brain tissues of PolySTAT-treated animals, with statistically significant reductions of 38% to 68% observed at the lesion surface and up to 400 μm from the lesion boundary (Fig. 3C; extravasation measurements for sham versus intact animals are reported in fig. S8B). Combined, these data demonstrate that intravenous PolySTAT reduces tissue hypoxia and preserves vascular integrity after TBI.

### PolySTAT enhances tissue sparing after TBI

**Fig. 4.**
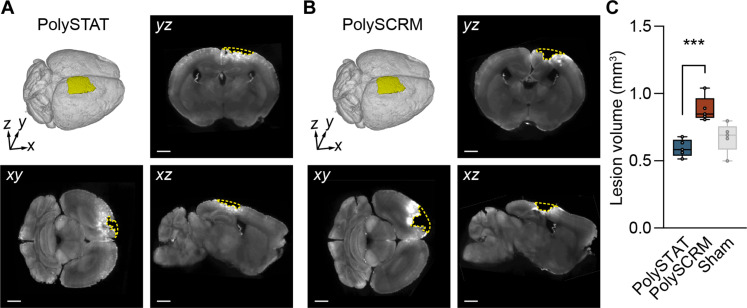
Effects of PolySTAT on brain tissue sparing. Lesion masks (yellow) are shown in both the 2D plane and 3D volume for one brain from each of the PolySTAT-treated (**A**) and PolySCRM-treated groups (**B**). Scale bar, 1 mm. (**C**) Box plots illustrate that PolySTAT-treated mice (*n* = 6) have smaller lesions (less tissue loss) compared to PolySCRM-treated mice (*n* = 6). Uninjured sham mice (*n* = 3) served as controls (statistical comparisons to PolySCRM-treated mice are not shown). ***P ≤ 0.001 by one-way ANOVA with Fisher’s LSD.

The extravasation of blood proteins into brain parenchyma after TBI causes loss of brain tissue at and near the injury site (*10*, *12*, *52*). Therefore, we used OTLS whole-tissue imaging, along with a computational filter (fig. S6), to quantify tissue loss (i.e., lesion volume) in the treated versus control groups (Fig. 4, A and B). Notably, lesion volume was reduced by 33% in PolySTAT- versus PolySCRM-treated TBI animals (Fig. 4C). Moreover, lesion volume in PolySTAT-treated animals did not differ significantly from sham animals (Fig. 4C; lesion measurements for sham versus intact animals are reported in fig. S9A), demonstrating that PolySTAT treatment increases brain tissue sparing after TBI.

### PolySTAT reduces the expression of inflammatory and gliosis genes after CCI-TBI

**Fig. 5.**
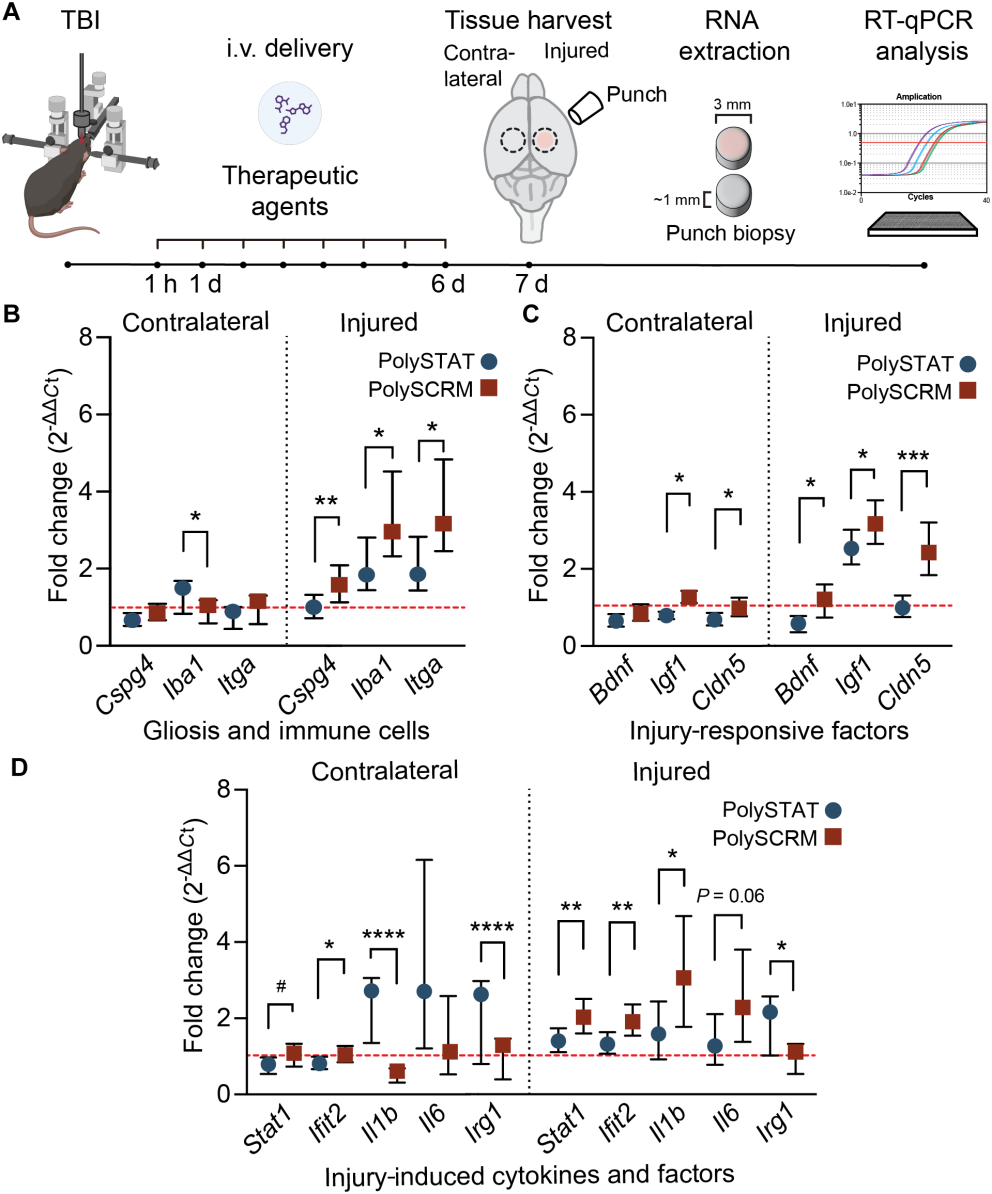
Effects of PolySTAT on the expression of genes that are markers of inflammation and gliosis. (**A**) RNA harvested from tissue biopsies of the contralateral and injured cortex (ipsilateral) of PolySTAT-treated (circles) or PolySCRM-treated (squares). Biopsy tissue was used for RT-qPCR. The red dotted line indicates 1× expression, and the difference in gene expression is analyzed by fold change (2^−∆∆*C*t^) versus uninjured sham biopsies. (**B**) Changes in the expression of astrogliosis genes *Gfap* and *Cspg4*, and immune cell genes *Iba1* and *Itga*. (**C**) Expression of injury-responsive growth factors *Bdnf* and *Igf1*, and tight junction *Cldn5*. (**D**) Changes in RNA expression of proinflammatory cytokine and fibrin-responsive genes *Stat1*, *Ifit2, and Il1b*, in addition to injury-induced cytokines *Il6* and *Il10*, and immuno-responsive gene *Irg1*. The data are presented with mean values indicated by a central symbol and SDs represented by error bars. ^#^*P* = 0.055; *P ≤ 0.05; **P ≤ 0.01; ***P ≤ 0.001; ****P ≤ 0.0001 by unpaired two-tailed *t* test. Created in BioRender. Han, Q. (2025) https://BioRender.com/272sw0h.

After injury, the central nervous system (CNS) is known to undergo a programmed response known as reactive gliosis, which is thought to mitigate injury and reduce further neural tissue damage and loss. Reactive gliosis ultimately results in the formation of a glial scar, which is known to affect the repair and regeneration of lost cells (*49*). While the mechanism that induces these reactive processes is not fully understood, the extravasation and deposition of blood proteins (i.e., fibrinogen/fibrin) have been shown to increase chondroitin sulfate proteoglycan deposition (e.g., *Cspg4*) in the extracellular matrix, astrocyte and microglia activation, and an influx of peripheral macrophages (*12*). Since PolySTAT treatment reduces vascular extravasation after TBI, we analyzed whether it similarly curtails the expression of genes associated with gliosis and inflammation, processes that contribute to neurodegeneration. Thus, we used real-time polymerase chain reaction (PCR) and analyzed gene expression in punch biopsies of uninjured (contralateral) and injured (ipsilateral) cortex taken from PolySCRM- and PolySTAT-treated animals 7 days post-injury (Fig. 5A). Notably, while glial fibrillary acidic protein (*Gfap*) expression trended downward, PolySTAT significantly reduced the expression of the reactive gliosis marker chondroitin sulfate proteoglycan (*Cspg4*) by 32%. Genes associated with microglia activation and signaling (allograft inflammatory factor; *Iba1*) decreased by 54%. Similarly, genes expressed by recruited monocytes and macrophages (integrin α; *Itga*) decreased by 60% (Fig. 5B), which indicates a reduction in reactive gliosis and the infiltration of peripheral blood–borne macrophages in the injured cortex.

Since PolySTAT improved the sparing of the neurovascular network and brain tissue, we hypothesized that it would also reduce the expression of genes that promote tissue growth and repair of the BBB. For example, the expression of brain-derived neurotrophic factor (*Bdnf*) is typically increased as a tissue repair response in the injured CNS (*53*). PolySTAT-treated animals had a significant attenuation of *Bdnf* mRNA levels by 35% in the injured cortex. Similarly, insulin-like growth factor (*Igf1*), which stimulates cell replacement after injury (*54*), was reduced by 36% with PolySTAT treatment. Moreover, the expression of an essential BBB tight junction protein (*55*), claudin-5 (*Cldn5*), was decreased 63% in PolySTAT-treated animals (Fig. 5C).

In addition to up-regulating reactive gliosis transcripts, vascular extravasation increases the expression of cytokines that promote inflammation and recruit immune cells to sites of CNS injury (*56*). Therefore, we analyzed the expression of signal transducer and activator of transcription (*Stat1*) and interferon-γ response factor (*Ifit2*), both of which have been shown to increase the proinflammatory responses of microglia and macrophages (*57*). PolySTAT reduced the expression of *Stat1* by 34% in the injured cortex and by 18% in the contralateral cortex. Similarly, PolySTAT reduced the expression of *Ifit2* by 33% in the injured cortex and by 15% in the contralateral cortex (Fig. 5D). Another proinflammatory cytokine, interleukin-1b (*Il1b*), was also reduced (64%) in the injured cortex. *Il1b* expression was significantly increased in contralateral tissue from PolySTAT-treated TBI animals, suggesting that *Il1b* could play a key role in mitigating tissue loss and vascular dysfunction (*58*). Similarly, *Il6* trended upward in the contralateral tissue of PolySTAT-treated animals. Both *Il1b* and *Il6* are thought to promote vasculogenesis after injury. Thus, the observation that both *Il1b* and *Il6* are down-regulated at the injury site in PolySTAT-treated animals could be reflective of the ameliorated vascular loss. Therefore, the altered pattern of gene expression also reinforces the observation by OTLS imaging that BBB function is improved with PolySTAT treatment, which reduces the local production of cytokines that are up-regulated in response to vascular injury in PolySCRM tissues. We observed less significant gene expression changes for other injury-induced cytokines in PolySTAT- versus PolySCRM-treated animals (fig. S10) (*59*, *60*). The expression of immunoresponse gene 1 (*Irg1*), which has been shown to be up-regulated by *Il1b* (*61*, *62*), was increased by 104% and 149% in the injured and contralateral cortex of PolySTAT-treated animals (Fig. 5B) and may reflect the role of an alternate TBI-induced modulator of Irg1 gene expression (*63*).

## DISCUSSION

Neurovascular networks are complex branched structures composed of vessels with varying diameters that culminate in a capillary network throughout the CNS. The damage and loss of neurovascular networks and the chronic dysfunction that occurs with TBI have been shown to correlate with chronic neurodegeneration and the onset of dementia (*10*). Currently, dexamethasone is the only clinically approved anti-inflammatory drug to treat TBI. While dexamethasone is thought to reduce edema after TBI, glucocorticoids have been shown to reduce neurovascular repair (*64*). The strategy of using a hemostatic polymer to cross-link and stabilize fibrin may appear counterintuitive, as perivascular fibrin accumulation at sites of BBB disruption is known to exacerbate inflammation and neurodegeneration post-injury (*65*, *66*). Yet, fibrin-binding platelet-like particles that increase coagulation have been shown to reduce microglia activation and tissue loss after TBI (*18*). By targeting the fibrin that leaks into the brain acutely after TBI, we hypothesize that PolySTAT acts as a molecular patch to stave off further neurovascular breakdown and loss, unlike current glucocorticoid therapies. Thus, we developed a 3D imaging and analysis pipeline to quantify anatomical features and structural changes in these complex neurovascular networks as a means for understanding how therapeutic interventions affect brain health.

While many approaches have been developed for whole-brain imaging and analysis, few studies have used multichannel high-resolution 3D analyses to quantitatively assess the effects of TBI on neurovascular integrity. Our study reports the development and first use of an unbiased 3D imaging and analysis pipeline to evaluate TBI in a way that accommodates the varying and unpredictable geometries of injured brains with minimal human intervention. We used fluorescent probes that label blood vessels, sites of BBB disruption (extravasation), and low-oxygen regions (hypoxia) to quantify whether intravenous PolySTAT affects neurovasculature and brain tissue loss following TBI. We used an OTLS microscope to image and reconstruct the 3D neurovascular network and to map each fluorescent tag at ~4-μm resolution throughout the whole mouse brain. We then developed a computational pipeline to accurately segment the lesion (region of tissue loss) in 3D, enabling us to quantify localized changes (adjacent to the injury site) that may otherwise be missed with whole-brain analysis (fig. S11, A to D). Our 3D microscopy and analysis pipeline allows for the quantification of multiple fluorescent labels within a nondestructive 3D volume, avoiding artifacts commonly associated with traditional histology (e.g., stretching, folding, or compression) and obviating the need for complex 3D reconstructions from tens or hundreds of serial sections (*21*, *67*, *68*). As a demonstration of the utility of our pipeline, we show that PolySTAT treatment preserves neurovascular networks and tissue after TBI, with high statistical confidence and reproducibility across multiple animals and diverse treatment conditions. In addition to evaluating therapeutic efficacy, our 3D imaging and analysis pipeline provides a valuable framework to assess how hemostatic interventions affect vascular function after TBI. Low-volume resuscitants and pro-coagulant agents remain an area of active investigation, as their use after TBI may reduce vascular flow or promote clot formation, which could further disrupt BBB repair and exacerbate reactive gliosis and neuroinflammation (*69*, *70*).

In previous studies, PolySTAT has only been used to bind fibrin and prevent deaths in a femoral bleed trauma model. Here, our 3D imaging and analysis pipeline demonstrated that PolySTAT increases brain tissue sparing and vascular stability. We aimed to corroborate the imaging data by quantitatively assessing whether PolySTAT reduced the expression of genes associated with neuroinflammation and reactive gliosis that occurs after TBI. We used the expression of gene markers (*Itga* and *Iba1,* respectively) as a proxy for changes in peripheral blood–borne macrophages and microglia infiltration, which correlates with the reduced blood extravasation and is quantified by our 3D image analysis. The reduced expression of proteoglycans (*Cspg4*) and tight junction proteins (*Cldn5*) correlates with increased neurovascular integrity and tissue sparing. The attenuation of blood extravasation and brain tissue loss would attenuate the need for growth factors (e.g., *Bdnf* and *Igf1*) that would promote further glial cell expansion and scar formation in untreated TBI animals. Similarly, the general downward trend in proinflammatory cytokine expression (highlighted by *Stat1*, *Il1b*, and *Il6*) provides additional evidence suggesting that intravenous PolySTAT preserves neurovascular integrity, which reduces inflammation associated with neurovascular damage and dysfunction after TBI. The expression of oxidative response gene *Irg1,* which has been shown to reduce ischemic injury (*71*), was increased significantly with PolySTAT treatment and correlated with reduced hypoxia and lesion volumes (as demonstrated by the OTLS analysis), and highlights the need for future studies to investigate the role of *Irg1* in mitigating hypoxic injury. Combined, these data suggest that PolySTAT forms a molecular patch to preserve neurovascular networks and reduce hypoxia and tissue loss after TBI. Future studies are thus needed to determine whether PolySTAT merely forms a barrier to prevent extravasation or whether it contributes to preserving the integrity of the neurovascular unit.

The analysis of whole-brain volumes at high resolution enabled us to precisely quantify and compare anatomical metrics with a degree of precision that traditional histology approaches cannot achieve. Moreover, our analysis pipeline does not rely on human bias to quantify the short-term benefits that fibrin cross-linking has on neurovascular function preservation in injured brains. Traditionally, the analysis of post-injury brain pathophysiology has been performed on thin tissue sections interspersed at 150- to 300-μm gaps, which are then used to sample and mathematically calculate volumes or population changes (*72*–*74*). Thus, by using a whole-brain analysis, our unbiased image analysis pipeline minimizes the introduction of human-sampling artifacts and errors that would increase variability and hamper the finding of significant results with traditional approaches. We demonstrate that acute administration of PolySTAT reduces the acute pathophysiology associated with blood vessel damage after a moderate CCI-TBI. Therefore, our 3D imaging and analysis pipeline can enable future studies that aim to accurately characterize whether PolySTAT mitigates the long-term tissue changes that occur after TBI, or whether PolySTAT preserves brain vasculature after a severe injury. Moreover, our 3D analysis approach can characterize how therapeutics affect neuron survival by tracking projection axons and neuronal circuit health across the brain to sites ≥5 mm away from the initial TBI site. The analysis pipeline also demonstrates the potential to characterize the spatial and cellular distribution of gene expression changes within computationally defined tissue regions. Thus, our 3D image analysis pipeline can be adapted to characterize how therapeutic interventions support the neurovascular unit and reduce neuroinflammation after TBI. Consequently, we have made our 3D image analysis tools available as an open-source package for other researchers studying brain injuries and other neurological conditions.

In summary, we delivered a fibrin-binding hemostatic polymer acutely after TBI to ameliorate neurovascular damage. We have developed an imaging and analysis pipeline that enables rigorous and localized 3D analyses and quantification of complex vascular and tissue metrics in whole mouse brains that exhibit unique injury patterns and organ-level morphologies after TBI. Based on this pipeline, we show that intravenous PolySTAT significantly reduces blood leakage and neural tissue loss after CCI-TBI. Moreover, our analysis shows that PolySTAT reduces neurovascular loss deep within the brain (up to approximately 1 mm from the lesion boundary). In particular, our 3D imaging and analysis pipeline provides the advantage of analyzing histological metrics with respect to the site of TBI (fig. S11, A to D). We further validated this 3D analysis by showing that intravenous PolySTAT reduces the expression of genes associated with inflammation and injury. Together, these studies demonstrate the potential utility of PolySTAT as an acute drug therapy for mitigating the cascade of vascular and cellular events that exacerbate brain injury.

## MATERIALS AND METHODS

### Synthesis and characterization of PolySTAT and PolySCRM

PolySTAT and PolySCRM were synthesized according to previously described methods by Chan *et al.* (*14*). Briefly, the polymer backbone was synthesized via reversible addition-fragmentation chain transfer with glycerol monomethacrylate (GmMA) and N-hydroxysuccinimide methacrylate (NHS-MA) monomers. The GmMA monomer was combined with 4-cyano-pentanoic acid dithiobenzoate (CTP) and 2,2′-azobis(2-methylpropionitrile) (AIBN) with a 40:160:1:0.333 NHSMA/GmMA/CTP/AIBN ratio in dimethylacetamide at a monomer concentration of 0.6 M. FBP was then grafted onto co-polymer backbone by the C-terminal lysine in the scrambled or FBP peptide in dimethyl sulfoxide (DMSO) with *N*,*N*-diisopropylethylamine (5× molar equivalents) at 50°C for 24 hours. Upon completion, unreacted NHSMA was capped with 1-amino-2-propanol (10× molar equivalents). Peptide co-polymer conjugates were then purified by extensive dialysis against phosphate-buffered saline (PBS) (*15*).

### Tissue preparation

Before TBI, animals were anesthetized and placed in a stereotactic frame. A craniotomy was performed to create a small burr hole (~1 mm diameter) approximately 1.0 to 1.8 mm caudal to bregma (1.0 mm medial-lateral) to allow placement of an injury probe (1 mm diameter) on the cortex, above the hippocampus. A fourth-generation Ohio State University impactor was placed on the dura with a touch force of 2 to 4 kDyn. CCI-TBI was induced by accelerating the probe dorsoventrally (4.3 m/s) to a displacement depth of −1.0 mm with a 15-ms dwell (*75*). After the contusion injury, the probe was retracted, and the bone deficit was repaired with bone wax. The scalp was closed, and the animal was allowed to recover with fluid and analgesic administration (0.05 mg/kg buprenorphine) in a heated environment. CCI-TBI was performed on early-adult female C57BL/6 mice (12 to 14 weeks old). To minimize variability arising from the neuroprotective effects of estrogen and progesterone after TBI (*76*–*78*), the mice were group-housed for 3 weeks after arrival. Animals were mixed and randomly assigned to treatment groups after injury. Additionally, each study cohort was injured on the same day, and each study was completed within one estrus cycle (i.e., ≤7 days). Post-injury, these mice were treated with PolySTAT or PolySCRM (nonbinding scrambled control polymer) intravenously (7.5 mg/kg) 1 hour post-injury. For mice used in real-time quantitative PCR (RT-qPCR) analyses, PolySTAT or PolySCRM was administered intravenously at 7.5 mg/kg per day at 1 hour, 24 hours, 2 days, 3 days, 4 days, 5 days, and 6 days post-injury and tissue was harvested 7 days post-injury. Each study included a sham group that underwent the craniotomy but did not receive a CCI-TBI, serving to isolate the effects of the surgical procedure alone. The intact control group neither underwent craniotomy nor received CCI, thereby maintaining normal brain function and serving as a baseline for comparison against the other groups. All animal procedures were approved by the Institutional Animal Care and Use Committee (IACUC) at University of Washington under protocol number 4053-01.

### Fluorescent biomarkers for analysis of neurovascular dysfunction

Fluorescent markers for vascular and BBB dysfunction were administered intravenously by retro-orbital injection. Three hours before euthanasia, each animal was injected with fluorescent dextran (70 kDa, Texas Red labeled, 125 μmol/kg, Thermo Fisher Scientific) and Image-iT hypoxia green (50 μmol/kg, Thermo Fisher Scientific). After euthanasia, Tomato-lectin Dylight 650 (Vector Labs, Thermo Fisher Scientific) was injected intracardially for vasculature labeling 3 to 5 min before saline perfusion and paraformaldehyde fixation. After dissection, each brain was optically cleared using a modified iDISCO procedure with final index matching in ethyl cinnamate (Millipore Sigma) (*25*, *79*).

### Light-sheet microscopy imaging

The cleared brains were imaged using the low-resolution mode of a hybrid multi-resolution OTLS microscope, achieving ~4-μm isotropic resolution (*43*). For each mouse brain, hypoxia signals and blood vessel signals were imaged using 488- and 638-nm excitation laser channels, with a multi-band band-pass filter (FF01-432/515/595/730-25, Semrock) on the collection path. Next, the extravasation signals (70-kDa dextran Texas Red) were imaged using the 561-nm laser channel, under a single-band band-pass filter (FF01-600/50-25, Semrock). Each dataset was stored in pyramidal HDF5 file format, and the metadata (channel information, tile positions) was stored in a separate XML file for stitching and reconstruction of each 3D volume for analysis.

### 3D dataset processing and analysis workflow

The datasets were stitched and fused using the BigStitcher plugin of ImageJ (*79*, *80*). This enabled us to extract 32× down-sampled datasets from the 561-nm laser channel (42.94-μm pixel spacing) for lesion analysis, 4× down-sampled datasets from both the 488- and 561-nm laser channels (5.37-μm pixel spacing) for hypoxia and extravasation analysis, and 2× down-sampled datasets from the 638-nm laser channel (2.68-μm pixel spacing) for blood vessel segmentation and analysis.

To determine the regions of interest, mouse brain datasets from the 561-nm laser channel were processed with a thresholding method to eliminate camera noise. The retained signals, including brain tissue autofluorescence and extravasation markers, were then used to extract the lesion boundary through a surface filtering algorithm (as implemented in the “Surface Wrap Solidify” extension of 3D Slicer) (*81*, *82*). Subsequent refinement was performed manually, focusing on confining the masks to the injury site within the right cerebral hemisphere. Following lesion boundary delineation, the lesion boundaries were uniformly expanded by a predefined margin using a spherical dilation operation, with each 100 μm corresponding to approximately 18 pixels (*82*). This expansion facilitated the generation of regions of interest masks at varying distances from the initial injury site (e.g., 0 to 100 μm and 100 to 200 μm). Because of the unique morphology of each lesion surface, the resulting masks varied across samples. These masks enabled the precise segmentation and subsequent extraction of regions of interest (conformal layers) for the analysis of hypoxia, extravasation, and blood vessel signals.

Within each defined region of interest (conformal layer), blood vessel signals were segmented using a Hessian-based Frangi vesselness filter (*44*) (as implemented in the “SlicerVMTK - Vesselness Filtering” extension of 3D Slicer). Following the segmentation process, key vascular features, including centerlines, radius, endpoints, and branch points, were extracted. From these features, comprehensive vascular metrics, including total vessel length and vessel volumes, were calculated. These metrics were subsequently normalized relative to the corresponding tissue volumes.

To accurately segregate hypoxia and extravasation signals, background-signal thresholds were established using the uninjured contralateral hemisphere, distal from the injury site. These thresholds separated hypoxia or extravasation signals (from exogenously delivered fluorophores) from inherent brain tissue autofluorescence. To minimize interindividual variability, thresholds for each brain were independently determined based on the 99.9th percentile of pixel intensity values in the uninjured hemisphere. Then, within each depth layer (from the injured ipsilateral hemisphere), the final quantitative metric was calculated by summing the volume above the signal threshold and normalizing it to the tissue volume within that layer.

For lesion masking, initially, the center of mass of the mouse brain volume was computed to define a central sagittal plane. The brain was then bisected along this plane into the left and right cerebral hemispheres. A mirrored 3D volume of the left hemisphere was subsequently generated. The volumes of the right (injured ipsilateral) hemisphere and the mirrored left (uninjured contralateral) hemisphere were registered and subtracted from one another to derive a 3D mask delineating the lesions in the right hemisphere. Finally, the lesion volume was quantified by summing all the voxels within the lesion mask.

The iDISCO clearing protocol typically results in tissue shrinkage of approximately 10%, as previously reported by Vigouroux *et al.* (*83*). To avoid the need for a correction factor for absolute tissue volume changes, we developed an analysis pipeline that does not require the 3D images to be skewed for registration with a brain atlas. Additionally, all quantitative comparisons were made between brains processed as a single batch, thus in parallel and with the same incubation times and protocol to ensure consistent tissue shrinkage among the specimens (fig. S9B).

### Tissue collection and processing for RT-qPCR

Twenty-four hours after the final polymer dose administration, mice were euthanized by lethal injection and then transcardially perfused with saline to exsanguinate the brain. Tissue samples were harvested from both the CCI-TBI lesion (ipsilateral) and contralateral cortex by placing a spatula under the dorsal surface of each cortex. A modified pipette was used to harvest a punch biopsy (~3 mm diameter) from each hemisphere. The collected tissue was placed in TRIzol and snap-frozen with liquid nitrogen before further processing or storage. Thawed tissues were homogenized in TRIzol with a tip sonicator on ice. RNA was isolated from each biopsy using the standard TRIzol manufacturer protocol, and 50 μg of total RNA was converted to cDNA by SuperScript IV reverse transcriptase (Invitrogen, standard protocol) and subsequently treated with RNase H (Invitrogen). Next, cDNA (50 ng) from each tissue sample was used with gene-specific primers (Table S1) in a SYBR Green master mix (Applied Biosystems, Thermo Fisher Scientific) for qPCR on a CXF Opus 384 (Bio-Rad). The critical threshold (*C*_t_) value was used to determine the expression level of each gene. The difference in target gene expression versus a common housekeeping gene [i.e., glyceraldehyde-3-phosphate dehydrogenase (GAPDH)] determined the ∆*C*_t_ for each animal. Next, the difference in ∆*C*_t_ values for PolySTAT- and PolySCRM-treated animals versus sham animals yielded the ∆∆*C*_t_, which was then used to calculate fold change (2^−∆∆*C*t^) for each target gene.

### Statistical analyses

The OTLS data for histological and biomarker metrics were preprocessed by removing outliers, defined as values beyond 1.5 times the interquartile range. Statistical significance for each metric was assessed using a one-way analysis of variance (ANOVA) to compare treatment groups with sham animals, using a significance level of *P* ≤ 0.05. Fisher’s least significant difference (LSD) test was applied for post hoc comparisons. The statistical significance for changes in specific target gene expression was determined by two-tailed unpaired *t* test. Significance was determined for *P* ≤ 0.05.
