# Supplementary material for: Delivery of a fibrin-binding hemostatic polymer ameliorates neurovascular damage and neural tissue loss after traumatic brain injury
Source: Sci Adv. 2025 Jul 18;11(29):eadw7425. doi: 10.1126/sciadv.adw7425 (PMC12273782; doi:10.1126/sciadv.adw7425)
Supplement: Supplementary file 1 — Figs. S1 to S11 Table S1 [file sciadv.adw7425_sm.pdf]

Science Advances
AAAS

Supplementary Materials for

Delivery of a fibrin-binding hemostatic polymer ameliorates neurovascular damage and neural tissue loss after traumatic brain injury

Qinghua Han *et al.*

Corresponding author: Drew L. Sellers, drewfus@uw.edu; Jonathan T. C. Liu, jonliu@uw.edu

Sci. Adv. **11**, eadw7425 (2025)
DOI: 10.1126/sciadv.adw7425

This PDF file includes:

Figs. S1 to S11
Table S1

Supplementary Figures

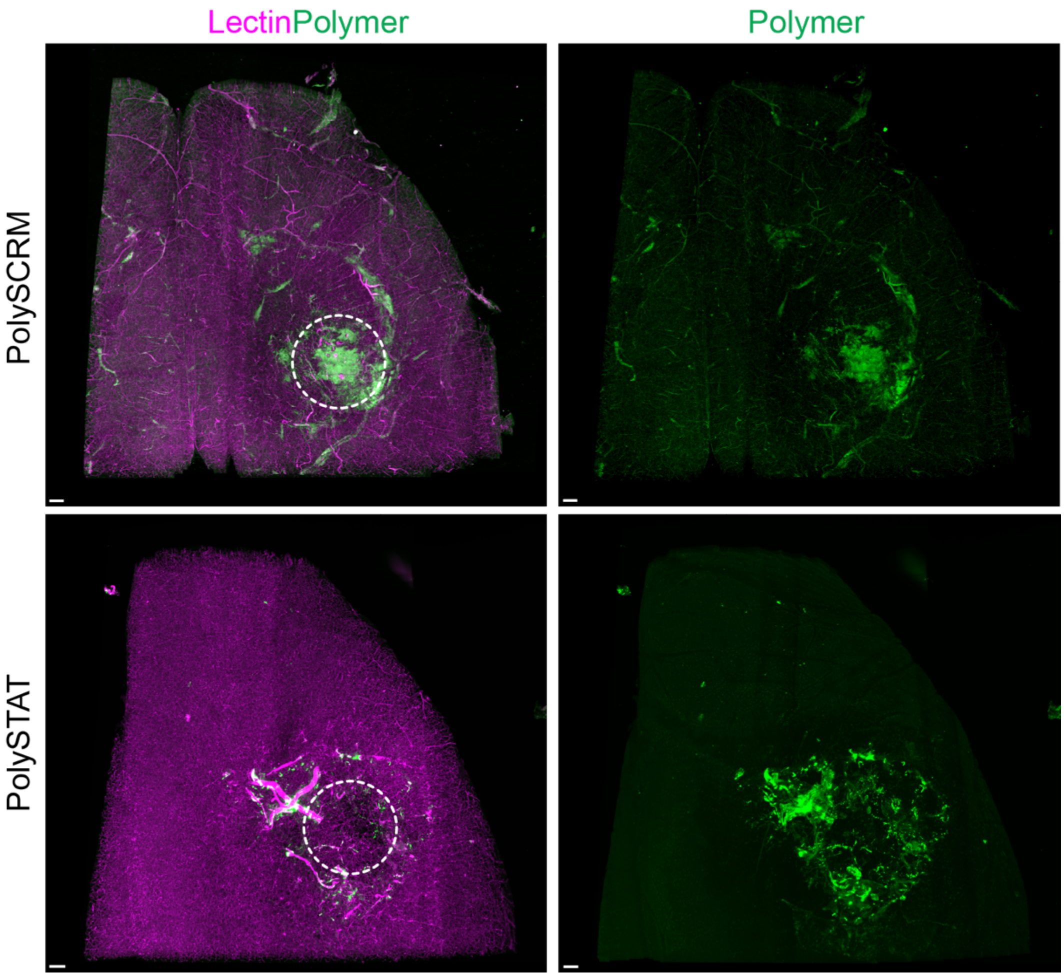

Fig. S1. Polymer distribution after CCI-TBI. 3D OTLS images of FITC-labeled PolySCRM and PolySTAT localization (green) in TBI tissue with tomato-lectin labeled blood vessels (magenta). The primary TBI lesion is highlighted by the circle. PolySCRM diffusely extravasates at the injury site whereas PolySTAT is localized to vessels and puncta within the lesion area. The brains were collected 6 hours after injury. Scale bar = 200 µm

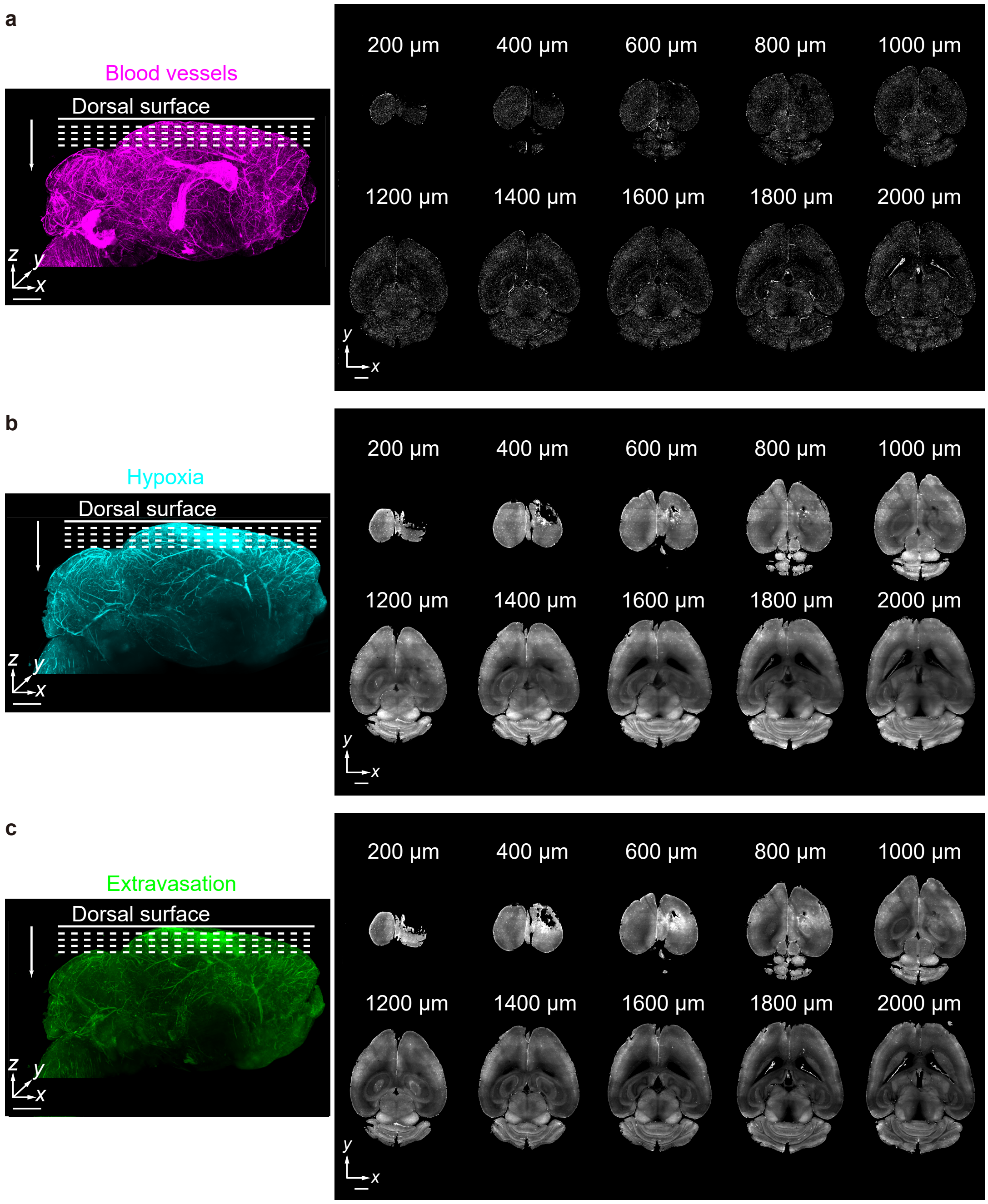

Fig. S2. Horizontal 2D slices across different imaging channels. Horizontal slices at 200 µm intervals (dotted white lines) from the dorsal surface of the brain in three channels: (**a**) blood vessels stained with tomato-lectin, (**b**) extravasation marked by texas red-labeled 70 kDa dextran, and (**c**) hypoxia labeled by a hypoxia green reagent. All scale bars = 1 mm.

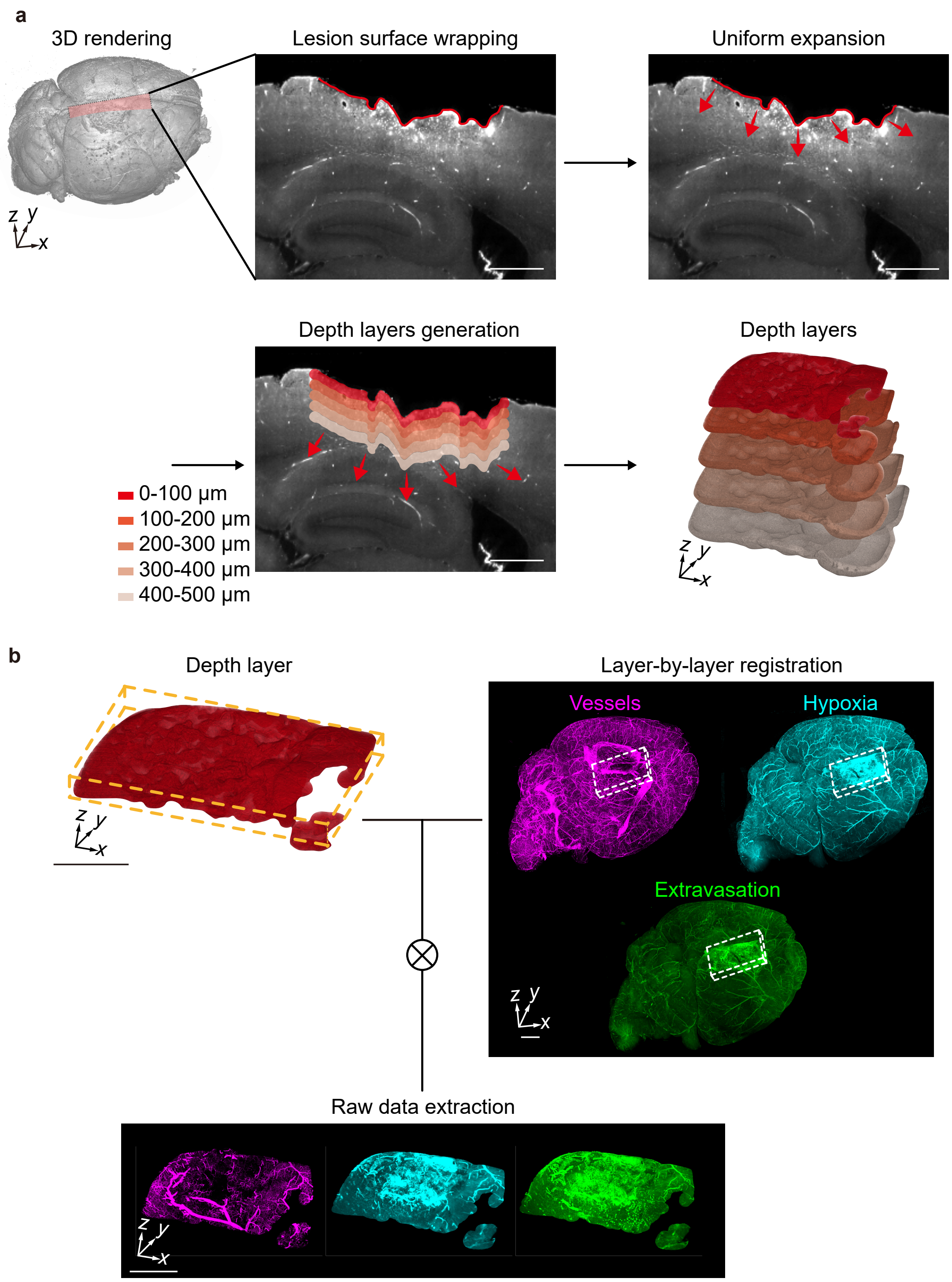

Fig. S3. Generation of depth layers. (**a**) A 3D rendered volume is generated from the autofluorescence signal in the extravasation channel (561 nm laser). The lesion surface is delineated (red line) using a surface-wrapping technique and is uniformly expanded to create depth layers (red arrows). Scale bar = 500 μm. (**b**) The depth layers are co-registered across all three channels. Data within each depth layer are extracted for subsequent segmentation and analysis. All scale bars = 1 mm in panel b. Panels (a) and (b) partially recapitulate data presented in Fig. 1c-d, providing additional details on the generation of depth layers and their layer-by-layer registration.

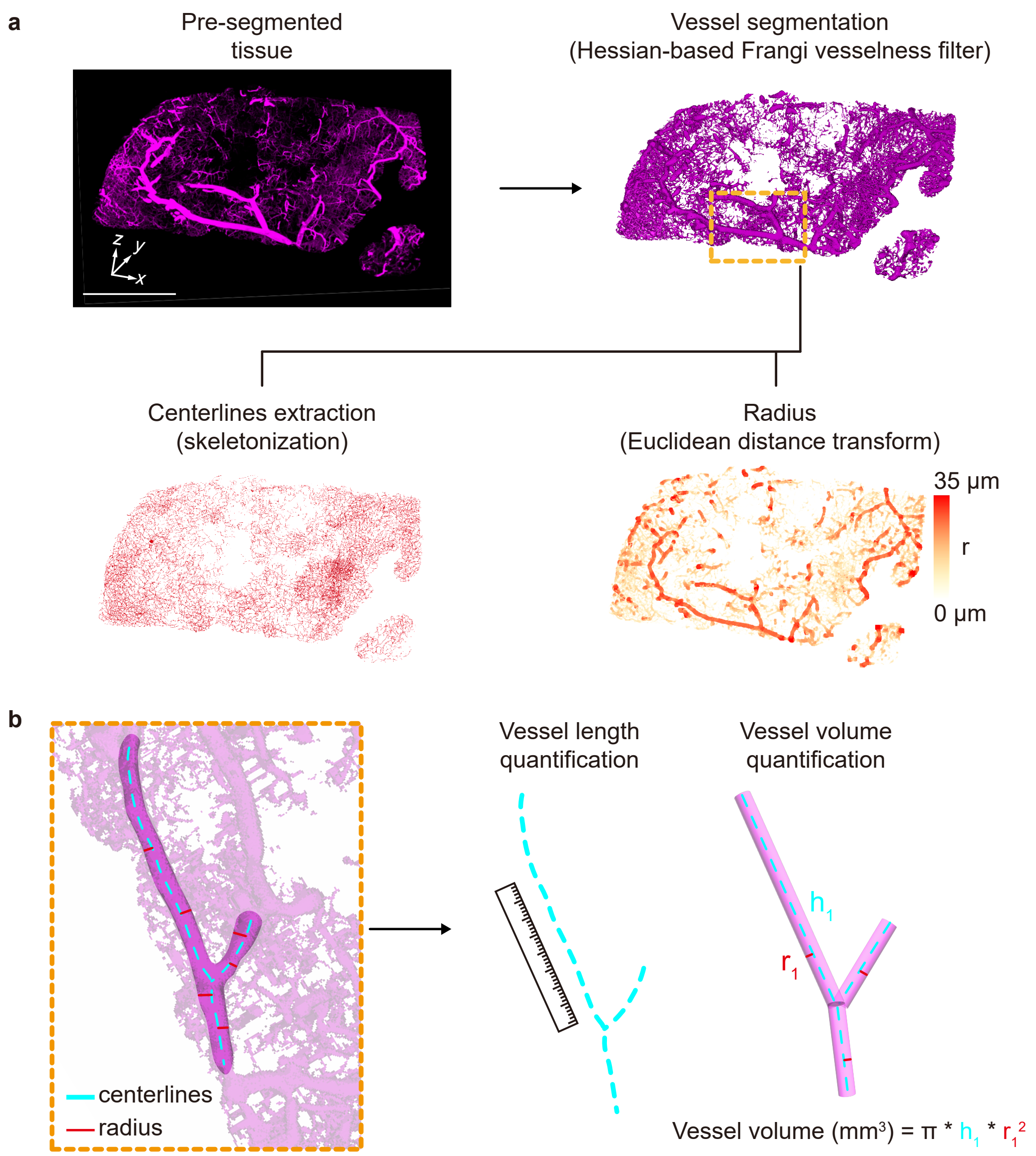

c

$$\text{Normalized vessel length (mm/mm}^3\text{)} = \frac{\text{Total vessel length (mm)}}{\text{Pre-segmented tissue volume (mm}^3\text{)}}$$

$$\text{Normalized vessel volume (mm}^3\text{/mm}^3\text{)} = \frac{\text{Total vessel volume (mm}^3\text{)}}{\text{Pre-segmented tissue volume (mm}^3\text{)}}$$

Fig. S4. Vessel segmentation and quantitative analysis. (**a**) Vessels in the raw dataset are segmented using a Hessian-based Frangi vesselness filter, followed by centerline extraction and the calculation of vessel radii. Scale bar = 1 mm. (**b**) The total vessel length is quantified by calculating the length of the centerlines (cyan). The total vessel volume is computed by approximating each vessel segment as a cylinder (magenta) and then using the centerline length and the average radius (r, red) for each segment. (**c**) Equations showing the normalization of total vessel length and total vessel volume (by dividing them with the tissue volume) to derive the final metrics.

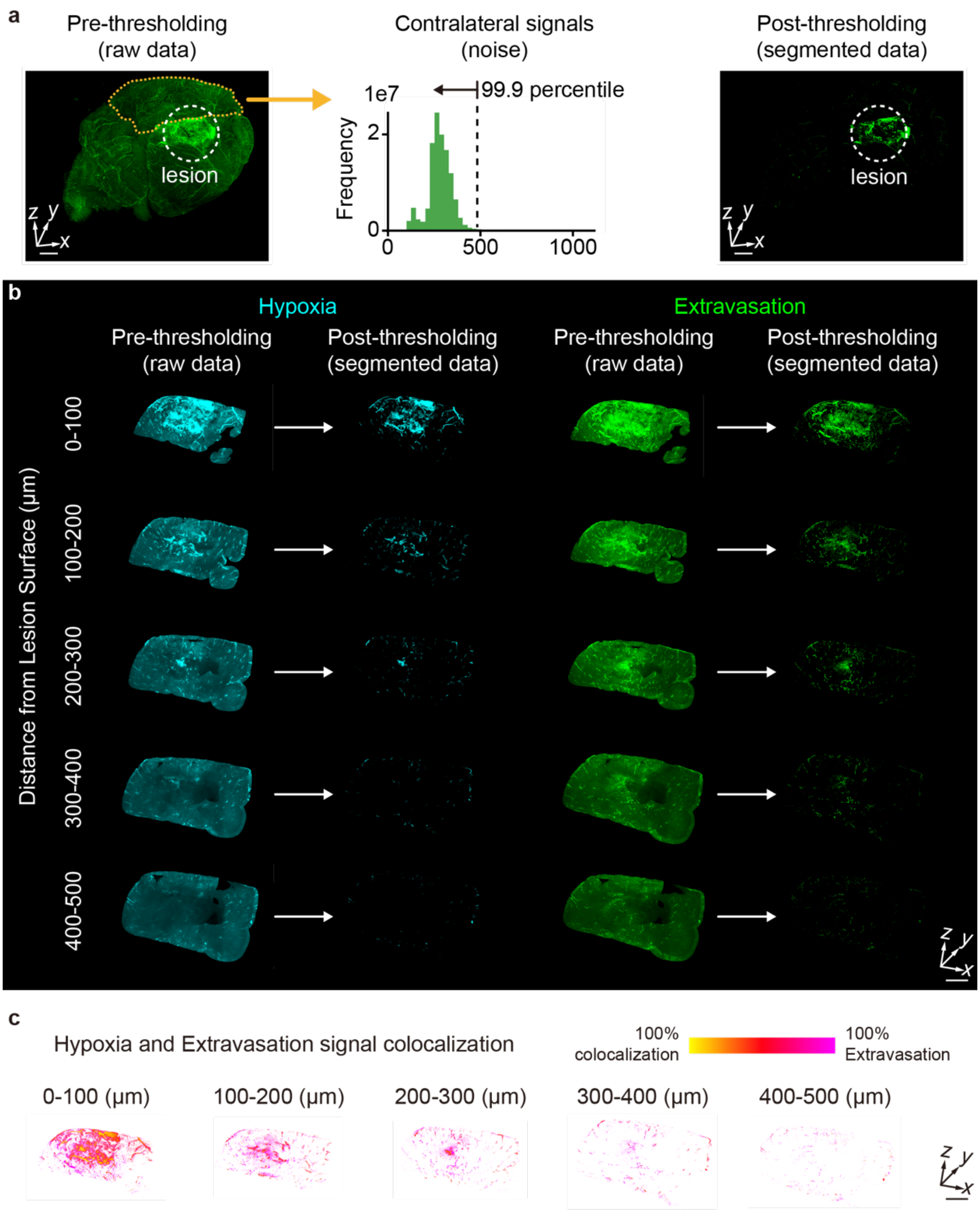

d

Post-thresholding volume (mm³) = Ipsilateral signal - Contralateral noise

$$\text{Normalized signal volume (mm}^3\text{/mm}^3\text{)} = \frac{\text{Post-thresholding volume (mm}^3\text{)}}{\text{Pre-thresholding volume (mm}^3\text{)}}$$

Fig. S5. Hypoxia and extravasation signal segmentation and quantitative analysis. (**a**) Background fluorescence levels from the left brain (i.e., the contralateral side in relation to the lesion) are used to determine the intensity thresholds for segmenting hypoxia and extravasation signals on the lesion-side of the brain. The thresholds are set at the 99.9th percentile of the contralateral-side intensity histogram for each brain specimen. Scale bar = 1 mm. (**b**) The thresholds are applied to each depth layer of a brain specimen

to segment hypoxia or extravasation signals. Scale bar = 1 mm. (c) Differential image analysis to evaluate the colocalization of hypoxia and extravasation signals within each segmented plane at distances ranging from 0 - 500 μm from the lesion surface. The colocalization heatmap reveals the transition from regions of complete colocalization (yellow) to areas with only dextran extravasation signals (magenta). Scale bar = 1 mm. (d) Total hypoxia volume and total extravasation volume are calculated by summing the voxels in the segmented regions. These values are then normalized by the total tissue volume (all voxels in the brain) to derive the final metrics.

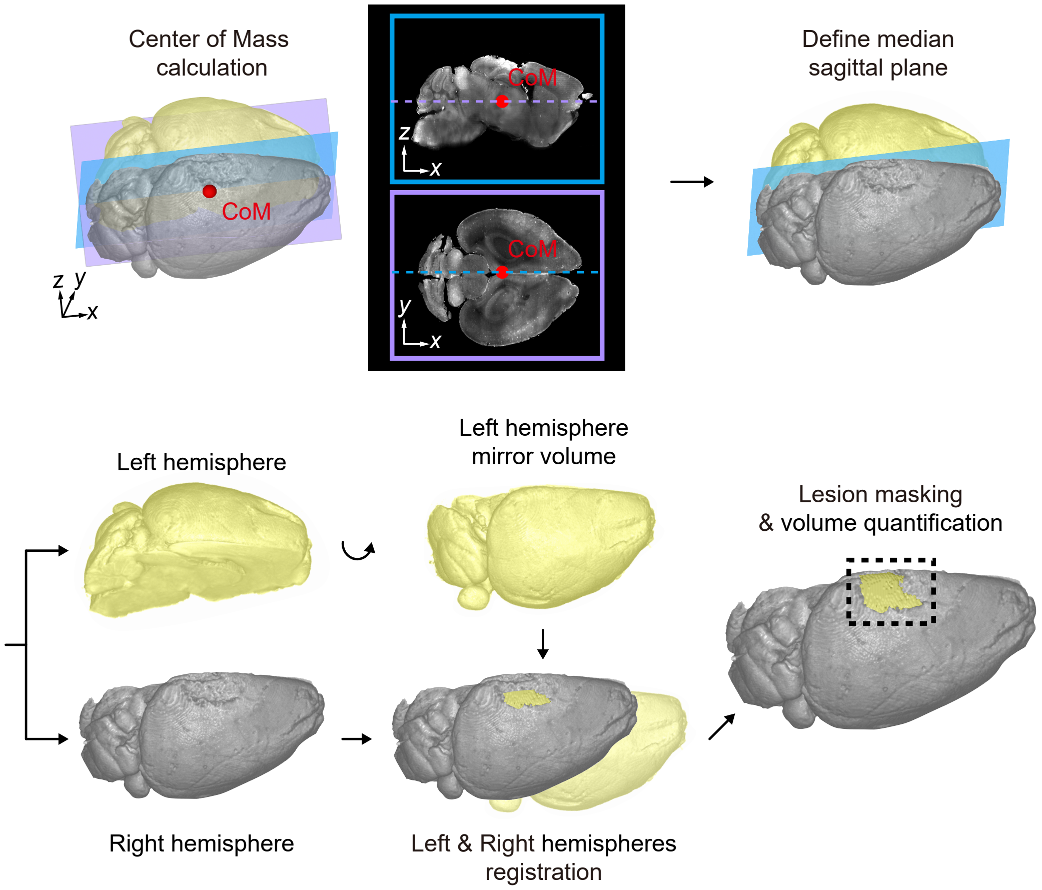

Fig. S6: Lesion masking and quantitative analysis. To approximate the (median) sagittal plane of the brain, the center of mass (COM) of the 3D rendered volume is calculated, dividing the brain into left and right hemispheres. Due to minor tissue asymmetries and the lesion on one side, the calculated COM is not perfectly symmetrical, necessitating additional registration for accurate analysis. A rigid transformation of a mirrored volume of the left hemisphere (contralateral side) is generated and then spatially registered in 3D to the right hemisphere (lesion side). The lesion is identified by overlaying the two hemispheres and segmenting the discrepant region, corresponding to the tissue void on the lesion side. The final lesion volume is computed by summing the voxels within the segmented lesion region.

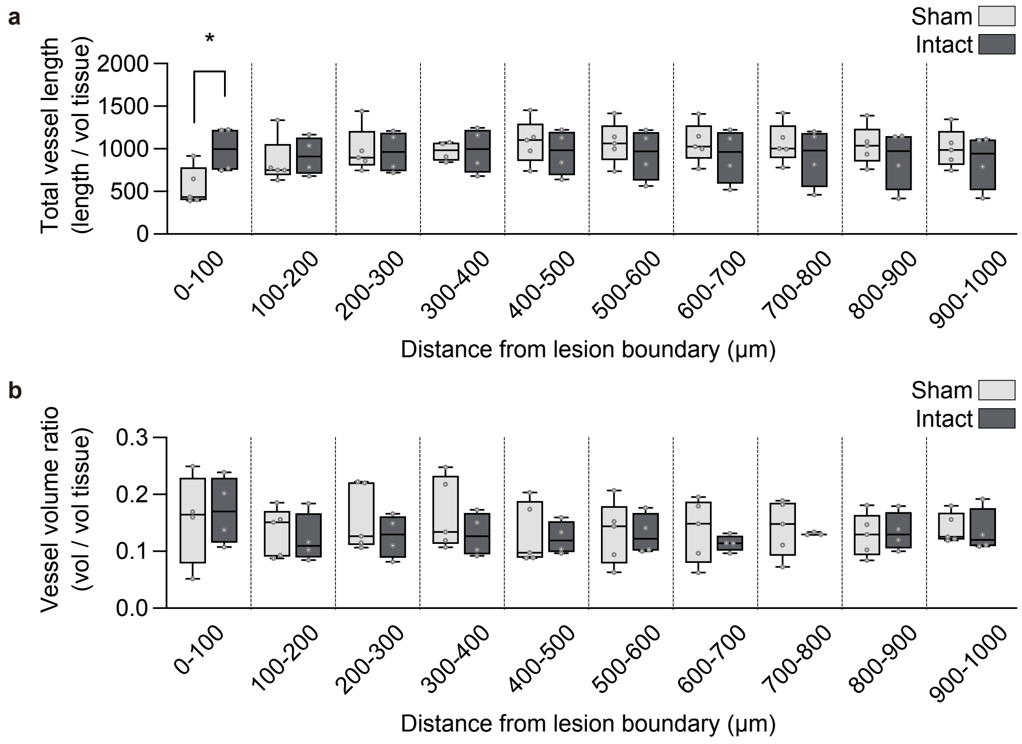

Fig. S7. Comparison of vascular metrics across groups. (**a**) Vessel length and (**b**) vessel volume compared between the Sham (n=5) and Intact (n=4) groups. *, $P \leq 0.05$ by one-way analysis of variance (ANOVA) with Fisher's least significant difference (LSD) test.

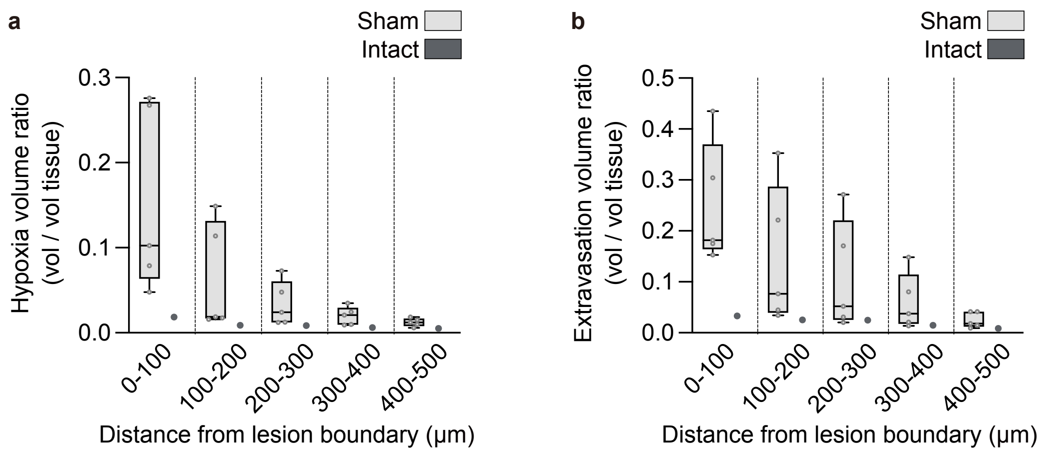

Fig. S8. Comparison of hypoxia and extravasation volumes. (**a**) Hypoxia volume and (**b**) extravasation volume compared between the Sham (n=5) and Intact (n=1) groups.

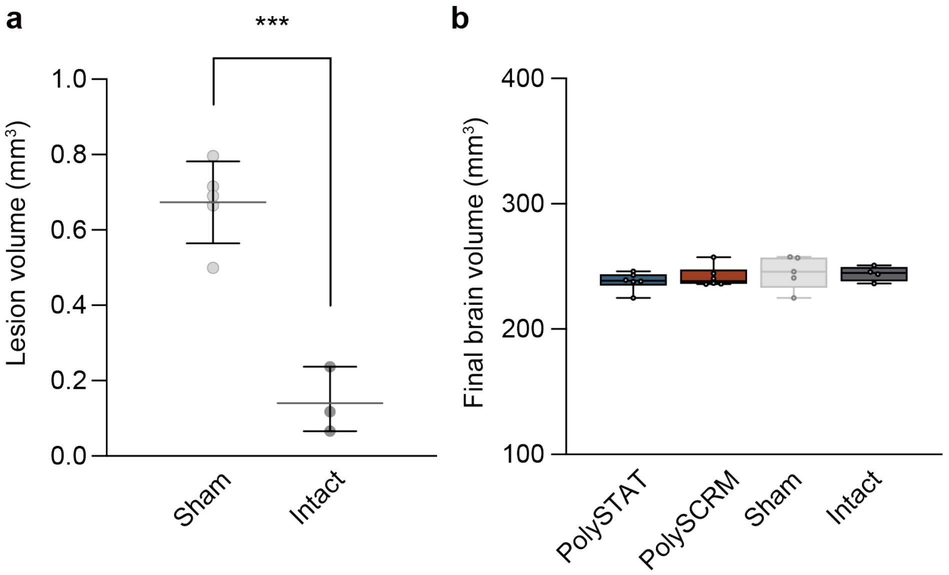

Fig. S9. Comparison of lesion volume. (**a**) Lesion volume compared between the Sham (n=4) and Intact (n=3) groups. ***, $P \leq 0.001$ by one-way analysis of variance (ANOVA) with Fisher's least significant difference (LSD) test. (**b**) Full brain volumes for PolySTAT, PolySCRM, Sham, and Intact groups after iDISCO processing.

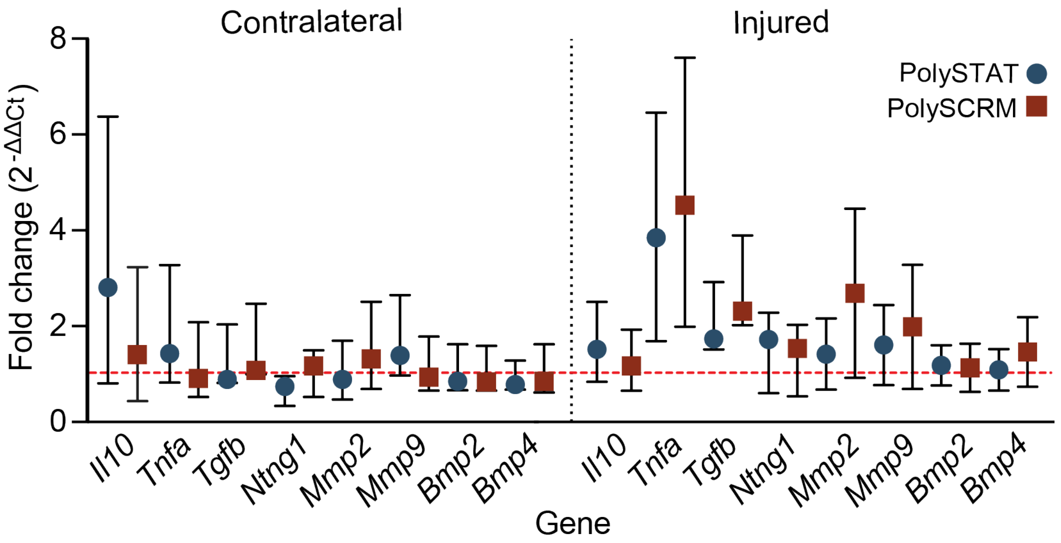

Fig. S10. PolySTAT effects on the expression of injury-induced genes. Real-time qPCR was performed on tissue biopsies harvested from the contralateral (grey background) and injured cortex from PolySTAT- (●) or PolySCRM-treated (■) TBI animals. The dotted red line indicates 1X gene expression levels in sham animals. Values are expressed as fold-change ($2^{-\Delta\Delta Ct}$) versus sham-control biopsies.

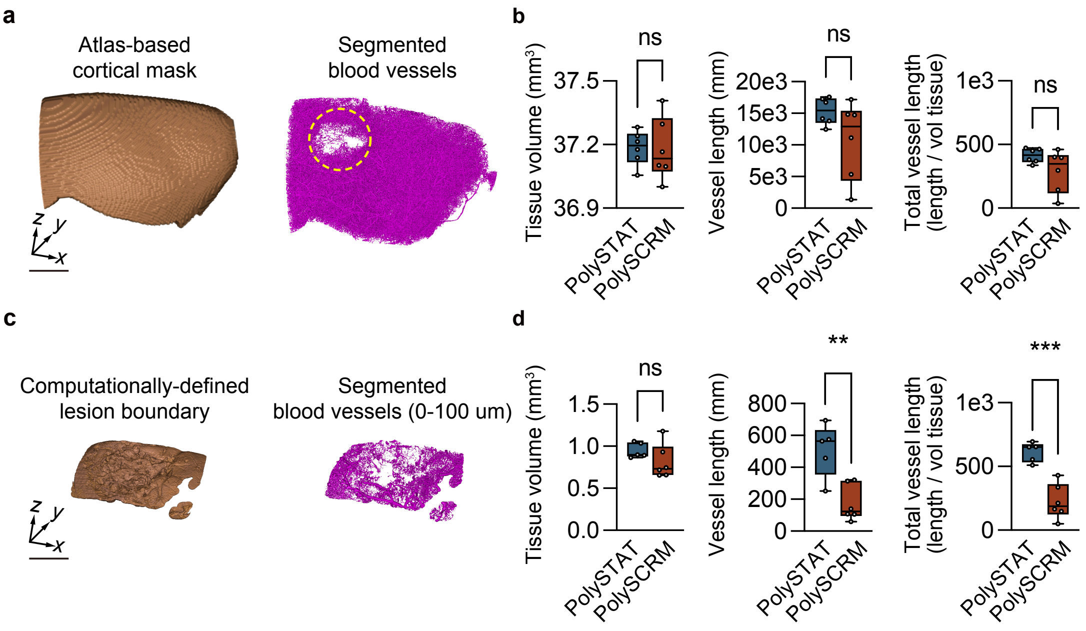

Fig. S11: Comparison of 3D vascular analyses using atlas-based cortical mask vs. depth-layer approach. (a) Whole-brain analysis using the atlas-based cortical mask shows no significant difference in vascular metrics (b), such as the vessel length ratio, between PolySTAT- (n=6) and PolySCRM-treated (n=6) animal models. In contrast, the depth-layer approach (c) analyzed localized regions and reveals statistically significant results (d). Data were analyzed using a t-test. ns, P > 0.05; **, P ≤ 0.01; ***, P ≤ 0.001. Scale bar = 1 mm. The yellow dashed region represents the lesion.

Supplementary Table

Table S1. Primers sets used for RT-qPCR analysis of gene expression in punch biopsy extracts.

Gene	PrimerBank ID	Primer	Sequence (5'-> 3')	Tm	Amplicon (bp)
BDNF	34328442a1	Forward	TCATACTTCGGTTGCATGAAGG	60.4	137
		Reverse	AGACCTCTCGAACCTGCCC	63	
Cldn5	31980735a1	Forward	GCAAGGTGTATGAATCTGTGCT	60.6	109
		Reverse	GTCAAGGTAACAAAGAGTGCCA	60.4	
CSPG4	20467423a1	Forward	GGGCTGTGCTGTCTGTTGA	62.2	132
		Reverse	TGATTCCCTTCAGGTAAGGCA	60.5	
GFAP	30692526a1	Forward	CGGAGACGCATCACCTCTG	62.1	126
		Reverse	AGGGAGTGGAGGAGTCATTCG	62.7	
Iba1	9506379a1	Forward	ATCAACAAGCAATTCCTCGATGA	60.3	144
		Reverse	CAGCATTCGCTTCAAGGACATA	60.7	
Ifit2	6680363a1	Forward	AGTACAACGAGTAAGGAGTCACT	60.2	172
		Reverse	AGGCCAGTATGTTGCACATGG	62.7	
Igf1	163659888c1	Forward	CACATCATGTCGTCTTCACACC	61.2	220
		Reverse	GGAAGCAACACTCATCCACAATG	61.9	
IL6	13624310c1	Forward	CTGCAAGAGACTTCCATCCAG	60.1	131
		Reverse	AGTGGTATAGACAGGTCTGTTGG	60.8	
IL1b	118130747c1	Forward	GAAATGCCACCTTTTGACAGTG	60.2	116
		Reverse	TGGATGCTCTCATCAGGACAG	61	
IL10	6754318a1	Forward	GCTCTTACTGACTGGCATGAG	60.2	105
		Reverse	CGCAGCTCTAGGAGCATGTG	62.7	
Irg1	950650a1	Forward	AATGAAACCTTGGGTCTTATGCC	60.8	170
		Reverse	TGCCCATGACTTATCCAGACAG	61.5	
ITGA	6680484a1	Forward	ATGGACGCTGATGGCAATACC	62.6	203
		Reverse	TCCCCATTCACGTCTCCCA	62.3	
Ntng1	9909140a1	Forward	TGCGAGGAAGGTGCAAGTG	62.5	288
		Reverse	CGCAGACATTAGCTTGTTTTGG	60.4	
PGLYRP1	6679293a1	Forward	GCCATCCGAGTGCTCTAGC	62.2	130
		Reverse	CTTGTGGTAATGCTGCACATTG	60.4	
STAT1	31543778a1	Forward	TCACAGTGGTTCGAGCTTCAG	62	155
		Reverse	GCAAACGAGACATCATAGGCA	60.4	
TGFb	6755775a1	Forward	CTCCCGTGGCTTCTAGTGC	62.1	133
		Reverse	GCCTTAGTTTGGACAGGATCTG	60.4	
TNFa	133892368c2	Forward	CTGAACTTCGGGGTGATCGG	62.3	122
		Reverse	GGCTTGTCACTCGAATTTTGAGA	61	
GAPDH	6679937a1	Forward	AGGTCGGTGTGAACGGATTTG	62.6	123
		Reverse	TGTAGACCATGTAGTTGAGGTCA	60.2	
